# Boosting cytotoxicity of adoptive allogeneic NK cell therapy with an oncolytic adenovirus encoding a human vIL-2 cytokine for the treatment of human ovarian cancer

**DOI:** 10.1038/s41417-023-00674-3

**Published:** 2023-11-10

**Authors:** D. C. A. Quixabeira, S. Pakola, E. Jirovec, R. Havunen, S. Basnet, J. M. Santos, T. V. Kudling, J. H. A. Clubb, L. Haybout, V. Arias, S. Grönberg-Vähä-Koskela, V. Cervera-Carrascon, E. Kerkelä, A. Pasanen, M. Anttila, J. Tapper, A. Kanerva, A. Hemminki

**Affiliations:** 1 Cancer Gene Therapy Group, Translational Immunology Research Program, Faculty of Medicine, University of Helsinki, Helsinki, Finland; 2 TILT Biotherapeutics Ltd, Helsinki, Finland; 3 Helsinki University Hospital Comprehensive Cancer Center, Helsinki, Finland; 4 Finnish Red Cross Blood Service, Helsinki, Finland; 5 Department of Pathology, University of Helsinki and Helsinki University Hospital, Helsinki, Finland; 6 Pathology, Finnish Food Authority, Helsinki, Finland; 7 Department of Obstetrics and Gynecology, Helsinki University Central Hospital, Helsinki, Finland

**Keywords:** Drug development, Cancer immunotherapy

## Abstract

Despite good results in the treatment of hematological malignancies, Natural killer (NK) cells have shown limited effectiveness in solid tumors, such as ovarian cancer (OvCa). Here, we assessed the potential of an oncolytic adenovirus expressing a variant interleukin-2 (vIL-2) cytokine, Ad5/3-E2F-d24-vIL2 (vIL-2 virus), also known as TILT-452, to enhance NK cell therapy efficacy in human OvCa ex vivo. Human OvCa surgical specimens were processed into single-cell suspensions and NK cells were expanded from healthy blood donors. OvCa sample digests were co-cultured ex vivo with NK cells and vIL-2 virus and cancer cell killing potential assessed in real time through cell impedance measurement. Proposed therapeutic combination was evaluated in vivo with an OvCa patient-derived xenograft (PDX) in mice. Addition of vIL-2 virus significantly enhanced NK cell therapy killing potential in treated OvCa co-cultures. Similarly, vIL-2 virus in combination with NK cell therapy promoted the best in vivo OvCa tumor control. Mechanistically, vIL-2 virus induced higher percentages of granzyme B in NK cells, and CD8+ T cells, while T regulatory cell proportions remained comparable to NK cell monotherapy in vivo. Ad5/3-E2F-d24-vIL2 virus treatment represents a promising strategy to boost adoptive NK cell therapeutic effect in human OvCa.

## Introduction

Natural killer (NK) cells are effector cells that are a part of the innate immune system and constitute important responders to viral infections in inflammatory processes [1, 2]. During neoplastic transformation, NK cells act by eliminating cancer cells from primary tumors and from metastatic sites through direct cancer cell lysis by secretion of granzyme B (GrzmB) and inflammatory cytokines [3, 4]. Unlike CD4+ and CD8+ T cells, NK cells do not express T cell receptors (TCR), and therefore NK cell’s mechanisms of cancer cell killing are not dependent on HLA I peptide presentation [1, 5]. Instead, NK cell activation is mediated by recognition of altered or lacking MHC I expression on malignant cells and co-expression of co-stimulatory and inhibitory receptors such as NKp46, NKp30, NKp44, NKG2D and DNAM1 [1, 4, 6, 7].

Because of its unique mechanism of action, recent interest has been raised for utilizing NK cells as an off-the-shelf candidate for allogeneic cell therapy in cancer treatment [5, 8]. This progress has been bolstered by improvement of methods for large-scale production of NK cell and expansion of NK cells from different cell sources, not only peripheral blood mononuclear cells (PBMCs). Indeed, generation of engineered NK cells options, like chimeric antigen receptor NKs (CAR-NK) and induced pluripotent stem cell (iPSC) [8–11] has expanded future options for NK cell therapy. Pre-clinical studies have shown promising tumor control in murine lymphoma tumors, and early-stage clinical trials have demonstrated good safety data in relapsed/refractory lymphoid malignancies [5, 8, 9]. Despite progress in hematological malignances, NK cell therapy efficacy in solid tumors is currently unimpressive.

A common pitfall for NK cell adoptive therapy success in solid tumors is its limited potential to circumvent immunosuppression in the tumor microenvironment (TME) [12]. Immunosuppressive tumors are defined by a milieu of tumor-associated cells, anti-inflammatory immune cells, and their derived products that drive immune evasion by outwitting immune-cells’ effector responses [12]. In this context, OvCa is an outstanding example of these features. Its microenvironment is often characterized by infiltration of immunosuppressive T regulatory (TReg) cells, myeloid-derived suppressor cells (MDSCs), and tumor-associated macrophages (TAMs) that have been proposed to contribute to therapy relapse and consequent tumor progression [13, 14]. In fact, this could partially explain the rapid tumor progression often associated with metastasis formation and high rates of tumor recurrence with poor 5-year survival in advanced cases [15].

In this context, oncolytic adenoviruses are emerging immunotherapeutic agents with known immunogenic capacity for mobilizing pro-inflammatory responses through selective lysis of infected cancer cells, shedding of tumor-associated antigens and epitope spreading in the TME [16]. In addition, adenoviruses are permissive to genetic engineering with inclusion of immunomodulatory genes into the virus construct leading to further therapeutic advantage [17].

Here, the virus used encodes a vIL-2 cytokine that selectively stimulates effector NK cells, CD4+ T, and CD8+ T cells while TReg cells remain unaltered [18]. The explanation for such effect relies on modifications made in the vIL-2 cytokine binding site to the wild-type IL-2 receptor (IL-2R) [19]. Variant IL-2 cytokine possesses higher binding affinity to the subunit IL-2Rβ (CD122), and with intermediate affinity to IL-2Rγ (CD132), while engagement with IL-2Rα (CD25) subunit is not necessary for its biological function [19–21]. In cancer immunotherapy this is relevant because TReg cells are the only lymphocytes in which stimulation depends on binding to the triple IL-2Rαβγ receptor [19]. Previously, we demonstrated that vIL-2 virus was able to deliver improved tumor response in an immunosuppressive hamster pancreatic model with over 60% complete responses as a monotherapy, and with a good safety profile [18]. Mechanistically, vIL-2 virus induced upregulation of granzyme genes and downregulation of MDSC-associated genes [18].

Recently, a study with oncolytic adenoviruses suggested that virus-infected OvCa cells induced NK cell cytotoxic function through modulation of DNAM-1 and TIGIT ligands on cancer cells [6]. This suggests a potential synergism when oncolytic adenoviruses are associated with NK cells in the context of cancer immunotherapy. In the present study, we propose the use of a genetically modified oncolytic adenovirus coding for a human variant IL-2 cytokine, Ad5/3-E2F-d24-vIL2 (aka TILT-452), to enable efficient NK cell therapy anti-tumor response in human OvCa tumors.

## Methods

### Surgical patient samples

Human cancer samples were collected from patients with confirmed diagnosis of OvCa (Table 1) who underwent surgical resection at the Hospital District of Helsinki and Uusimaa (HUS). Specimens from twelve OvCa patients were received in 10% FBS RPMI-1640 media, and processed upon arrival into single cell suspension following a previously optimized protocol [22]. OvCa samples were stored at −140 °C until further analysis.

**Table 1:** Patient characteristics and diagnosis information.

Patient ID	Age	Diagnosis	Specimen resection site	Primary tumor location	Prior cancer treatments
HUSOV1	57	HGSC^a^ Stage IIIB	Ovary	Fallopian tube	–
HUSOV2	76	HGSC Stage IVB	Greater omentum	Fallopian tube	–
HUSOV3	75	HGSC Stage IVB	Greater omentum	Fallopian tube	Carboplatin+paclitaxel
HUSOV4	79	HGSC Stage IVB	Greater omentum	Fallopian tube	–
HUSOV5	76	HGSC Stage IVB	Greater omentum	Fallopian tube	–
HUSOV6	35	LGSC^b^ Stage IVB	Greater omentum	Ovary	–
HUSOV9	50	HGMEC of ovary (clear cell and endometrioid). Stage IC2	Ovary	Left ovary	–
HUSOV10	65	HGSC Stage IVB	Greater omentum	Left ovary	Carboplatin + paclitaxel
HUSOV12	73	HGSC Stage IVB	Greater omentum	Fallopian tube	Carboplatin
HUSOV13	66	HGSC Stage IVB	Greater omentum	Ovary	–
HUSOV15	79	HGSC Stage IVB	Ovary	Right ovary	Carboplatin
HUSOV16	62	HGSC Stage IVB	Greater omentum	Fallopian tube	Carboplatin+paclitaxel

Partial data was published as Quixabeira et al. [27] at ESMO Immuno-Oncology 2022.

^a^High grade serous carcinoma.

^b^Low grade serous carcinoma.

### Oncolytic adenoviruses

Construction of genetically modified oncolytic adenovirus Ad5/3-E2F-d24, here also referred as virus backbone, has been previously described [17]. To generate the oncolytic adenovirus coding for a human variant IL-2 protein, Ad5/3-E2F-d24-vIL2 (vIL-2 virus), also known as TILT-452, five-point mutations in the wild-type human IL-2 sequence at positions L80F, R81D, L85V, I86V and I92F were made. Then, the vIL-2 transgene was inserted under the E3 promoter replacing gp19k and 6.7k gene of the virus backbone construct [18]. Both viruses have the “delta24” deletion in E1a and an E2F promoter, rendering the virus highly specific to tumor cells aberrant in the Rb/p16 pathway, which is a universal characteristic of cancer cells. The virus has also been deleted for E1b19k, giving additional tumor specificity [23].

### Generation of allogeneic NK cells for adoptive cell therapy

To produce NK cells used in the experiments, human PBMCs were isolated from buffy coats of healthy blood donors (Finnish Red Cross Blood Service, Finland) with Lymphoprep gradient density separation (StemCell technologies, USA). NK cells were activated and expanded following the human NK cell activation/expansion kit (130-094-483, Miltenyi Biotec, CO, DE). Subsequently to 18 days of expansion, NK cells were isolated with the human NK cell isolation kit (130-092-657, Miltenyi Biotec) and freshly used in all in vitro and in vivo assays. Selection of donor for the allogeneic NK cell therapy to be used in all experiments was performed upon NK cell expansion from several PBMCs donors and subsequent screening for cytotoxicity with real time impedance system (Supplementary Fig. 1). The PBMCs donor selection was based on NK cell potential to kill OvCa cells. Here, donor 18 was selected.

### Real time co-culture cytotoxic studies

To assess the Ad5/3-E2F-d24-vIL2 virus potential to enhance NK cell adoptive therapy efficacy in the treatment of OvCa samples in vitro, cell cytotoxicity response was monitored in real time with the impedance system xCELLigence Real-Time Cell Analysis (RTCA) DP instrument (Agilent, CA, USA). All 12 OvCa human samples were seeded in duplicates at the concentration of 5 × 10^4^ cells per well into pre-coated impedance plates (E-Plate 16, 300601150, Agilent, CA, USA) with 5 µg/ml of human fibronectin (ECM001, Sigma Aldrich, MI, USA). After 24 h, freshly isolated and expanded allogeneic NK cells were added in 1:8 effector to target ratio (E:T) to the plates, and 100 vp/cancer cell of Ad5/3-E2F-d24-vIL2 or Ad5/3-E2F-d24 were added to the co-cultures. Negative controls were appropriately used for each condition.

### Immune studies of OvCa co-cultures

To study the changes on the lymphocyte cell compartments in OvCa ex vivo cultures treated with vIL-2 virus and NK cell therapy, flow cytometry was performed upon sample availability. OvCa samples were plated in triplicates and treated with allogeneic NK cells therapy in 1:8 (E:T) ratio in combination with Ad5/3-E2F-d24-vIL2 virus or Ad5/3-E2F-d24 (100 vp/cancer cell). Cells were harvested after 5 days of co-culture and lymphocytes stained for flow cytometry analyses. Intracellular staining was performed with BD GolgiPlug™ containing Brefeldin A (555028, BD, NJ, USA) and cell permeabilization with BD Cytofix/Cytoperm™ Plus Fixation/ Permeabilization Kit (555028, BD, NJ, USA), performed according to manufacturer protocols. For the staining of transcription factor, cells were processed according to the True-Nuclear™ Transcription Factor Buffer Set (424401, BD, NJ, USA) protocol. Cell fluorescence was acquired with NovoCyte Quanteon Flow Cytometer Systems (Agilent, CA, USA), upon acquisition of 90k to 100k events per well. Cell gating and data processing were performed with FlowJo v.10.6.1 (FlowJo LLC, OR, USA). A list of all antibodies used in the present study can be found in Supplementary Table 1.

### Study of virus infection immune-modulation of NK cell ligands in OvCa cells

Changes on the expression of co-stimulatory and co-inhibitory receptors on the cancer cells surface for NK cell response were studied upon vIL-2 virus infection. Human OvCa samples were plated in triplicates (2 × 10^5^ cells/well), and after 24 h of incubation, samples were infected with Ad5/3-E2F-d24-vIL2 or Ad5/3-E2F-d24 virus, 100 vp/cancer cell. After 48 h, cells were harvested, stained, and analysed by flow cytometry as previously described. A list containing all the antibody fluorochrome-conjugated details are included in Supplementary Table 1.

### PDX OvCa in vivo animal experiment

The OvCa cell line used as a PDX tumor model was generated from a resected tumor fragment of a metastatic high-serous carcinoma stage IIIC patient, and the details for its development have been described elsewhere [24]. For the in vivo humanization of immunodeficient mouse, blood from the same patient was collected and PBMCs isolated with Lymphoprep gradient density separation. Due to limited cell availability, patient PBMCs were expanded for 14 days using the “young” TILs protocol [24, 25]. Expanded PBMCs were stored at −140 °C, and 24 h prior to animal injection PBMCs were rested in 6-well G-Rex plates with TILs media [22].

Patient-derived cancer cells were engrafted (3.5 × 10^6^ cells/animal) in 28 immunodeficient female NOD.Cg-PrkdcscidIl2rgtm1Sug/JicTac (Taconic Biosciences GmbH, Leverkusen, DE), 5–10 weeks old, subcutaneously in the animal’s left lower back. Animals were randomized into one of the experimental groups (seven mice per group) when ovarian cancer PDX tumors reached ~5–6 mm in the longest diameter. Intratumoral virus injections with 1 × 10^9^ vp/tumor of Ad5/3-E2F-d24 or Ad5/3-E2F-d24-vIL2 virus were given on experiment days 0, 3, 6, and 9. The virus treatment dose and optimal therapeutic scheme have been described previously [17, 24, 26]. On day 1, mice received a single injection with 1 × 10^7^ allogeneic NK cells intraperitoneally (i.p.). Treatment response was evaluated through tumor progression measured with a digital calliper every two days. Tumor volume was obtained through the formula (length × width^2^)/2, and percentage tumor growth was calculated by normalizing the values to their respective day 0 volumes. Of note, the investigators were not blinded to the group allocation during the treatments. The experiment was finished on day 12, and organs were harvested for histopathological analysis. Blood was collected, erythrocytes lysed with ACK buffer and white blood cells frozen down at −80 °C. In addition, tumors were harvested, processed into single cell suspensions with a tissue homogenizer, and frozen down at −80 °C. Flow cytometry was performed with the collected samples, following the same protocols described in previous sections. A list of antibodies used can be found in the Supplementary Table 1.

### Immunohistochemistry of human OvCa tumors and histopathology of mouse tissues

For histopathological studies, smaller fragments of received OvCa tumors were processed to histopathological analyses [22]. Slides were stained with hematoxylin and eosin (HE) and immunohistochemistry (IHC) was performed with antibodies targeting CD4, CD8, CD56, and PD-L1 expressing cells (Supplementary Table 2). OvCa slides were analyzed by an experienced pathologist following a commonly used clinical semi-quantitative scoring system for tumor-infiltrating lymphocytes distribution in tumors. Digital scans of slides were taken using 3DHISTECH Pannoramic 250 FLASH II digital slide scanner. PDX OvCa tumors and organs (heart, lung, liver, kidney, and spleen) from the mice in vivo study were collected on day 12 and processed as previously described [27]. HE slides from tumors and mouse tissues were analysed by a veterinary pathologist in a blind manner.

### Statistical analysis

Statistical interpretation of the flow cytometry and ex vivo studies was assessed by unpaired *t*-test with or without Welch’s correction. The normality of the tumor growth data was evaluated with Shapiro-Wilk test. Tumor control difference was evaluated by Two-way ANOVA with post-hoc Tukey correction. All statistical tests and graphical representation of the data were done with GraphPad Prism v.8.4.2, (GraphPad Software Inc, CA, USA).

## Results

### Set of OvCa patient tumors shows marked presence of tumor-infiltrating lymphocytes (TILs)

**Fig. 1:**
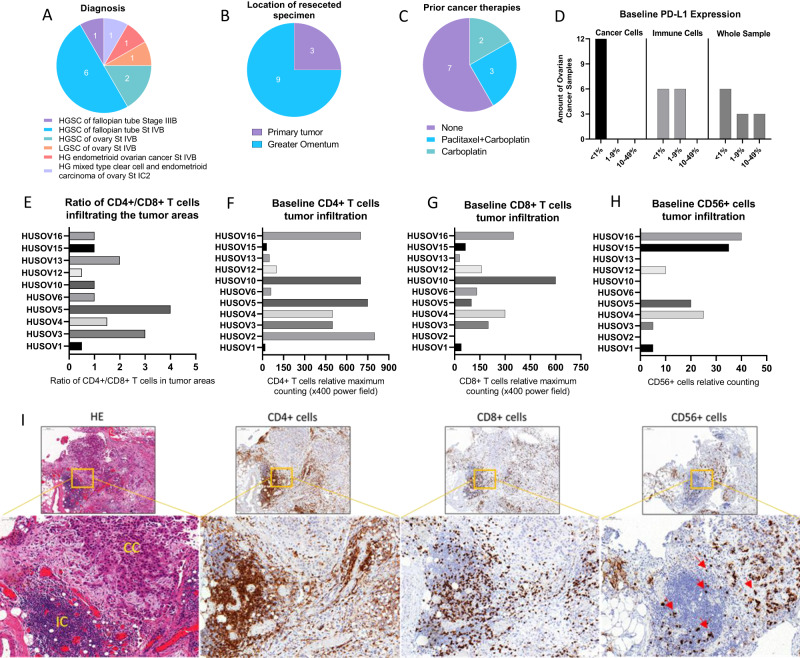
Ovarian cancer evaluation of baseline immune-status by immunohistochemistry and samples diagnosis characteristics. Upon arrival, fragments of ovarian cancer samples were fixed and embedded into paraffin blocks, and staining with HE and immunohistochemistry for CD4 + T, CD8 + T, CD56+ and PD-L1+ cells were performed. **A**–**C** Chart graphs detailing ovarian cancer characteristics on **A** diagnosis, **B** location of resected specimen, and **C** prior cancer therapies. **D** Expression of PD-L1 percentages levels on cancer cells, immune cells and overall counting in ovarian cancer samples. **E** Ratio of CD4+/CD8+ T cell infiltration across study samples. **F** Baseline maximum counting of CD4+ T cell infiltration across all ovarian cancer tumors in x400 power field. **G** Baseline maximum counting of CD8+ T cell infiltration across all ovarian cancer tumors in ×400 power field. **H** Baseline relative counting of CD56+ infiltrating lymphocyte present in each ovarian cancer samples. **I** Photos of slides representing lymphocytic infiltration in an ovarian cancer sample. From left to right, HE staining showing in yellow cancer cells (CC) and immune cells (IC) grouping, CD4+ T cells (brown), CD8+ T cells (brown), and CD56+ cells (red arrows) distribution in the same tumor area.IHC photos from HUSOV16 slides were used to exemplify the lymphocytic infiltration pattern. Upper row ×26 magnification (scale bar 200 µm) and lower row ×33 magnification (scale bar 100 µm). Partial data was published as Quixabeira et al. [27] at ESMO Immuno-Oncology 2022.

Samples included in the study comprised a diverse range of OvCa subtypes (Fig. 1A), and a majority of specimens collected were from metastatic lesions (Fig. 1B). Five patients had received neoadjuvant chemotherapy prior to surgery, while the remaining patients were naïve to cancer treatments (Fig. 1C). Immunohistochemistry results showed that all samples were nearly negative (<1%) for PD-L1 expression in cancer cells and half of the samples had some expression (1–9%) of PD-L1 in immune cells (Fig. 1D). Ratio analysis of CD4+/CD8+ T cell in tumors (Fig. 1E) indicated that OvCa samples were predominantly infiltrated by CD4+ T cell (Fig. 1F) compared to CD8+ T cell at the baseline (Fig. 1G). CD56+ lymphocytes were encountered in much lower number compared to CD4+ and CD8+ T cell, and their infiltration varied across samples with negative cell counting in 4 out 11 samples (Fig. 1H). Figure 1I, represents the histological lymphocytes infiltration findings in an OvCa sample.

### Ad5/3-E2F-d24-vIL2 virus improves NK cell therapy cytotoxicity in human OvCa tumor ex vivo co-cultures

**Fig. 2:**
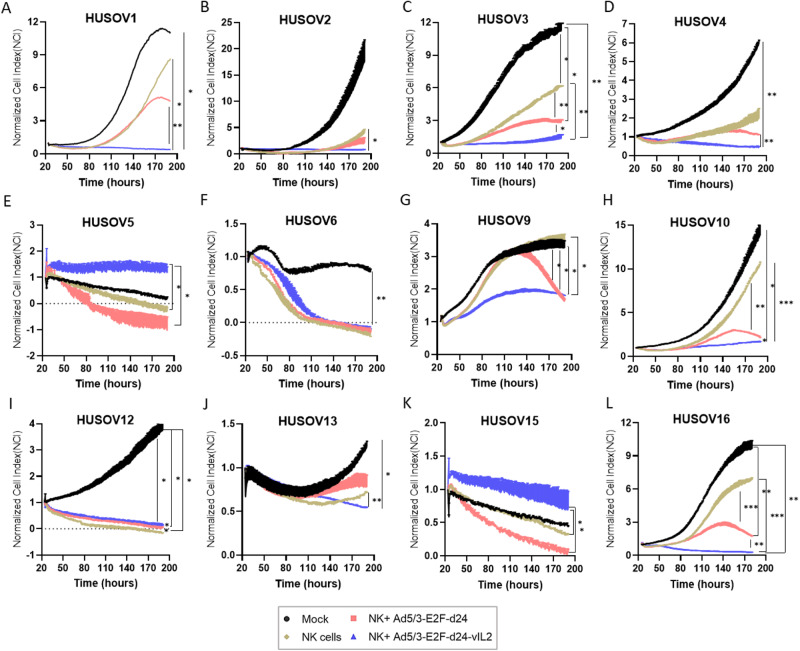
Cytotoxic effect of Ad5/3-E2F-d24-vIL2 plus adoptive NK cell therapy in human ovarian cancer tumor digests. **A**–**L** Individual real time responses of ovarian cancer samples treated with vIL-2 virus in combination with allogeneic adoptive cell therapy. Ovarian cancer human samples were seeded in duplicates at the concentration of 5 × 10^4^ cells per well into pre-coated impedance plates with 5 µg/ml of human fibronectin. Cells were incubated at 37 °C for 24 h, when freshly isolated and expanded allogeneic NK cells were added in 1:8 effector to target ratio (E:T) to the plates. Concurrently, Ad5/3-E2F-d24-vIL2 or Ad5/3-E2F-d24 virus treatments (100 vp/cell) were added to the co-cultures. Untreated samples digests were used as a mock and NK cell monotherapy as a control groups for the assay. Cytotoxicity effect in co-cultures was evaluated through plate scans every 15 min for a total of 190 h. Normalized cell index to each cell index time-point was used as the final readout for the assay. Data sets were analysed for statistical significance by unpaired *T* test and presented as mean ± SEM. *p < 0.05, **p < 0.01, ***p 0.001.

To assess the potential of the vIL-2 virus as an enabler of NK cell therapy, we treated OvCa tumor digests with allogeneic NK cell therapy and infected co-cultures with vIL-2 virus. Treatment response was measured by cell impedance in real time (Fig. 2A–L). Co-cultures treated with backbone virus plus NK cells had statistically significant better cancer cell killing potency than NK cell monotherapy in four out of 12 samples (HUSOV3, HUSOV9, HUSOV10, and HUSOV16) (Fig. 2C, G–H, L). Furthermore, consistent improvement of NK cell cytotoxicity was observed when vIL-2 virus was used in combination. Statistically significant higher cytotoxicity results were achieved in seven out of 12 samples (HUSOV1, HUSOV2, HUSOV3, HUSOV9, HUSOV10, HUSOV13, and HUSOV16) compared to NK cell therapy only (Fig. 2A–C, G, H, J, L). In HUSOV6 and HUSOV12, NK cell monotherapy and virus combined treatments had equal efficacy, however, statistical difference was observed between NK monotherapy and virus combined groups in HUSOV12 (Fig. 2F, I). Virus backbone plus NK cells and NK cell therapy had better results in cancer control than vIL-2 virus plus NK cells only in HUSOV5 and HUSOV15 (Fig. 2E, K). Of note, vIL-2 virus combination with NK cell therapy provided statistically significant better results compared to its backbone counterpart in HUSOV1, HUSOV3, HUSOV4, HUSOV10, and HUSOV16 (Fig. 2A, C, D, H, L).

### vIL-2 virus treatment potentiates NK+ cells immune reactivity in human OvCa co-cultures

**Fig. 3:**
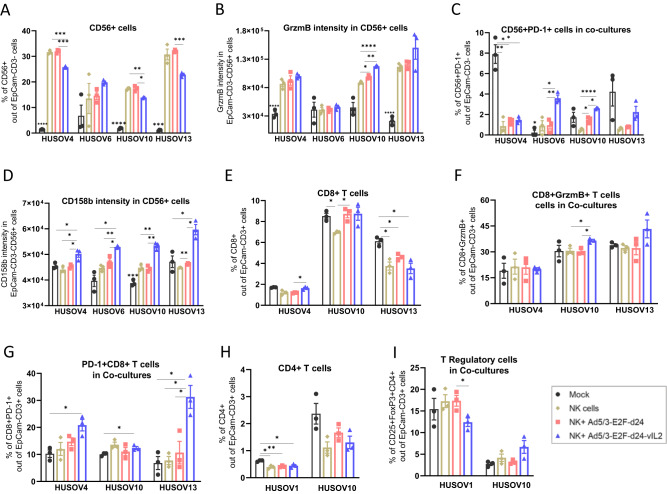
Immune status of lymphocytes present in human ovarian cancer co-cultures treated with combination therapy. HUSOV4, HUSOV6, HUSOV10, and HUSOV13 patient samples were seeded in triplicates at the concentration of 3.5 × 10^5^ cells per well. After 24 h incubation period at 37 °C, ovarian cancer samples were treated with allogeneic NK cells therapy in 1:8 (E:T) ratio in combination with Ad5/3-E2F-d24-vIL2 virus or Ad5/3-E2F-d24 (100 vp/cell). Mock control samples respective to each treatment condition were studied. Co-cultures were incubated for 5 more days, then cells were harvested and lymphocytes stained with antibody flurochrome-conjugated for flow cytometry analyses. **A** Levels in percentage of NK+ cells. **B** GrzmB mean fluorescence intensity (MFI) in NK+ cells. **C** Percentage of PD-1+ NK cells. **D** CD158b MFI in NK cells. **E** CD8+ T cells percentage levels. **F** GrzmB MFI in CD8+ T cells. **G** Percentage of PD-1 + CD8+ T cells. **H** Percentage of CD4+ T cells. **I** Levels of TReg+ cells in percentage. Data sets were analysed for statistical significance by unpaired *T* test with Welch´s correction and presented as mean ± SEM. *p < 0.05, **p < 0.01, ***p < 0.001, ****p < 0.0001.

Considering the immunostimulatory nature of vIL-2, we studied the consequences of NK cell plus vIL-2 virus therapy in effector NK+ cells in tumor co-cultures. On day 5, statistically significant higher percentages of NK+ cells were detected following NK cell monotherapy (HUSOV4 and HUSOV10) and backbone plus NK cells (HUSOV4, HUSOV10, and HUSOV13) therapies relative to vIL-2 plus NK cells (Fig. 3A). However, a different scenario was encountered when NK cytotoxicity was analyzed through GrzmB MFI in NK+ cells. Statistically significant higher levels of NK+ cells producing GrzmB were detected in HUSOV10 treated with vIL-2 virus plus NK cells (*p* < 0.05) than in other treatment groups (Fig. 3B). Likewise, a similar trend was observed in HUSOV4 and HUSOV13, although not statistically significant. Regarding the percentage of NK+ cells expressing programmed cell death protein 1 (PD-1), statistically significant higher levels of these cells were detected in HUSOV6 and HUSOV10 in vIL-2 virus plus NK cell therapy compared to the other tested treatments, while in HUSOV4 mock group had the highest percentage (Fig. 3C). However, the percentage of PD-1 + CD56+ cells did not seem to be directly changed by the viruses monotherapy infection in the studied co-cultures (Supplementary Fig. 2). Interestingly, vIL-2 virus in combination with NK cells significantly increased CD158b intensity in NK+ cells in all tested samples compared to nearly all other groups (Fig. 3D).

### CD8+ T cells cytotoxicity increases while frequency of T regulatory cells in OvCa co-cultures remains unchanged after treatment with vIL-2 virus and NK cell therapy

Additionally, we evaluated the treatment repercussions on the TILs present at the baseline in patient co-cultures. In the samples analyzed, levels of CD8+ T cells fluctuated regardless of the treatment used across all groups with significance noted (*p* < 0.05) between vIL-2 virus plus NK cells and virus backbone plus NK cells (HUSOV4), mock versus NK cell monotherapy, and virus backbone plus NK cells versus NK cell monotherapy (HUSOV10), and mock compared to all other groups in HUSOV13 (Fig. 3E). When assessing CD8+GrzmB+ T cells, we observed a trend towards cytotoxicity in HUSOV13 and HUSOV10, with the latter being statistically significant (*p* < 0.05) in the vIL-2 virus plus NK cell group compared to the other NK cell therapy receivers (Fig. 3F). Moreover, CD8 + PD-1+ T cells were found in statistically significant higher levels (*p* < 0.05) in vIL-2 virus combined with NK cell therapy in HUSOV4 and HUSOV10 (versus mock) and in HUSOV13, relative to all other groups (Fig. 3G). Levels of tumor-infiltrating CD4+ T cells were only significantly higher in HUSOV1 compared to the other groups (Fig. 3H). Interestingly, TReg cells in co-cultures were not changed by vIL-2 virus treatment in the samples checked compared to the other groups, except in HUSOV1 where backbone virus plus NK cells TReg cells percentage was statistically significant higher than in the vIL-2 virus combined group (*p* < 0.05) (Fig. 3I).

### Infection by vIL-2 virus does not facilitate immune evasion to NK cell response in OvCa tumors

**Fig. 4:**
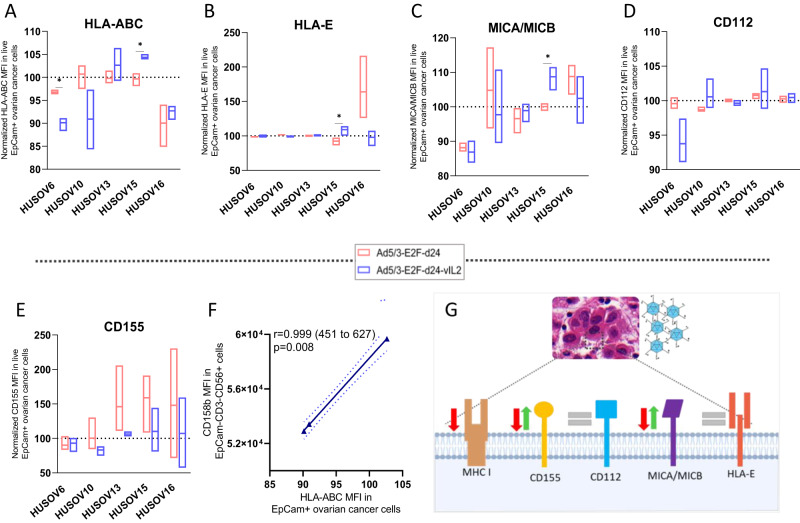
Ad5/3-E2F-d24-vIL2 modulation of expression of proteins associated to NK cell engagement in ovarian cancer cells. Differences on the expression of co-stimulatory and co-inhibitory proteins on the cancer cells surface for NK cell response were studied upon vIL-2 virus infection. Human ovarian cancer samples were plated in triplicates (2 × 10^5^ cells/well), and after 24 h of incubation, samples were infected with Ad5/3-E2F-d24-vIL2 or Ad5/3-E2F-d24 virus, 100vp/cell. Cells were harvested after 48 h, and cancer cells were stained with live/dead cell dye (PI), EpCam, CD112, CD155, MICA/MICB, HLA-ABC, and HLA-E surface markers and cell fluorescence analysed by flow cytometry. Results were normalized to their respective mock (uninfected) group and presented as normalized percentages. **A** HLA-ABC, **B** HLA-E, **C** MICA/MICB, **D** CD112, and **E** CD155 MFI in ovarian cancer cells from patient samples HUSOV6, HUSOV10, HUSOV13, HUSOV15, and HUSOV16. **F** Correlation between the expression of CD158b MFI in NK+ cells and HLA-ABC MFI in ovarian cancer cells. **G** Schematic summarizing vIL-2 virus mediated changes on the expression of key ligands for NK cell response. Data sets were analysed for statistical significance by unpaired *T* test with Welch´s correction and presented as floating bars (min to max). *p < 0.05.

Considering the role NK cells exert controlling viral infections [10], it was relevant to understand how vIL-2 virus modulates key ligands on the cancer cell surface that could compromise its synergism with NK cell therapy. Here, analysis of OvCa cells in tumor digests suggest a downregulation in the intensity of HLA-ABC expression in vIL-2 virus infected samples, as HUSOV6, HUSOV10 and HUSOV16 showed lower levels of HLA-ABC MFI compared to mock group (Fig. 4A). When infected by the virus backbone, intensity of HLA-ABC barely changed in HUSOV10, HUSOV13, and HUSOV15. In HUSOV16 and HUSOV6, HLA-ABC was slightly downregulated, with the latter being significantly higher than vIL-2 virus (*p* < 0.05). Overall, HLA-E intensity remained unchanged for both viruses studied (Fig. 4B). Curiously, MICA/MICB intensity varied similarly in both viruses used with no specific trend being observed in the analyzed samples, HUOSV15 being the only one with significant higher intensity in vIL-2 virus than in backbone (Fig. 4C). Intensity of CD112 in OvCa samples did not oscillate regardless of the virus used in all samples, only a minor downregulation was observed in HUSOV6 infected by vIL-2 virus (Fig. 4D). In addition, a trend towards upregulation of CD155 intensity in backbone-infected group was observed, while in vIL-2 virus group a variation on the expression prevailed (Fig. 4E). Lastly, we obtained a strong positive correlation between the HLA-ABC MFI expression in OvCa cells and CD158b MFI in NK cells from the co-cultures cytotoxicity assay (Fig. 4F). Summary of our findings are illustrated in Fig. 4G. Percentage analysis of studied markers can be found in Supplementary Fig. 3.

### Adoptive NK cell therapy provides efficient tumor control in vivo when combined with Ad5/3-E2F-d24-vIL2 treatment

**Fig. 5:**
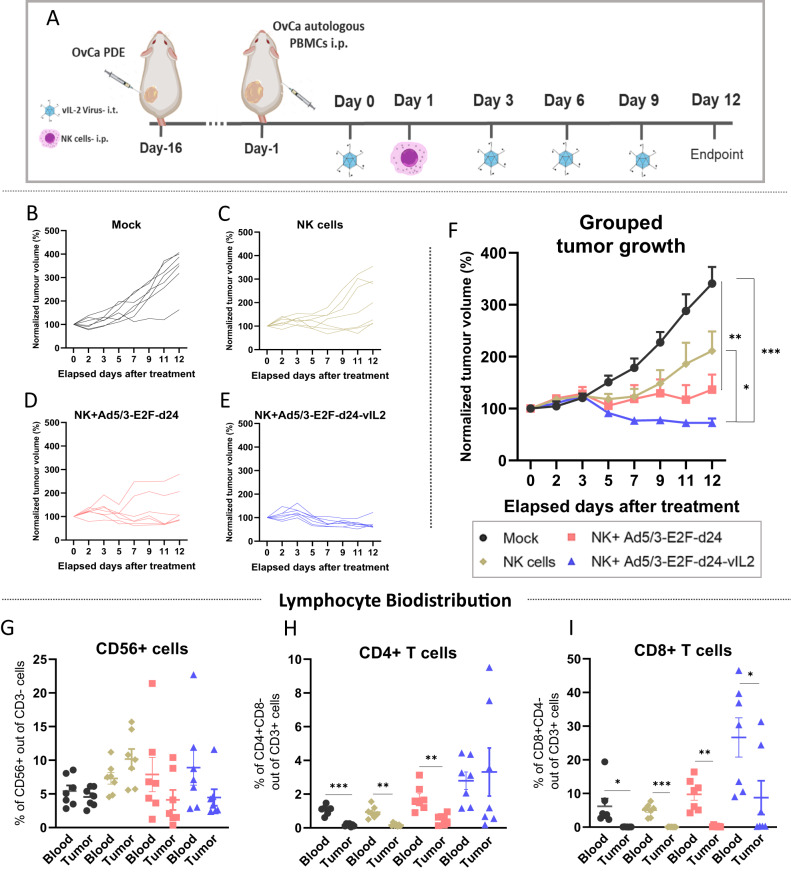
Ovarian cancer PDX in vivo model tumor response to vIL-2 virus plus NK cell therapy. Experiment started when tumors length reached 5–6 mm, on day 15 after tumor engraftment, animals were randomly assigned into one of the experimental groups, 7 animals per group, and the patient´s expanded PBMCs (5 × 10^6^ cells/ animal) were given via intraperitoneal injection. Virus intratumoral injections (1 × 10^9^ vp/tumor) with Ad5/3-E2F-d24 or Ad5/3-E2F-d24-vIL2 virus were given on experiment days 0, 3, 6, and 9. On day 1, mice received a single injection with 10 × 10^6^ allogeneic NK cells via intraperitoneal injection. A group receiving NK cell therapy only and an untreated mock group were used as controls in the experiment. To simulate the mechanical dissociation promoted by local virus injections, control animals were injected intratumorally with PBS on the same days of virus treatments. Experiment was finished on day 12. **A** Experimental design of the in vivo ovarian cancer PDX experiment. Individual normalized tumor growth in response to **B** mock, **C** allogeneic NK cell adoptive cells only, **D** Ad5/3-E2F-d24 plus NK cell, and **E** Ad5/3-E2F-d24-vIL2 plus NK cell therapies. **F** Combined tumor progression in response to therapies. **G**–**I** Blood and tumor percentage levels of **G** NK+ cells, **H** CD4+ T cells, and **I** CD8+ T cells in in mice across treatment groups. Combined tumor growth statistical significance was analysed by Two-way ANOVA and bar graphs by unpaired *T* test with Welch’s correction and presented as mean ± SEM. *p < 0.05, **p < 0.01, ***p < 0.001.

In order to validate our ex vivo findings in an in vivo pre-clinical setting, we performed an animal experiment utilizing an OvCa PDX model. When tumors had established, immunodeficient mice were humanized with the patient´s expanded immune cells intraperitoneally. Intratumoral virus treatments and NK cell therapy were administrated as described in the experimental design (Fig. 5A). The expression of vIL-2 transgene by the Ad5/3-E2F-d24-vIL2 virus in the OvCa PDX cell line was confirmed in vitro utilizing a previously described RT-qPCR assay (Supplementary Fig. 4) [18]. As displayed in the individual tumor growth curves, the untreated mock control group tumors grew rapidly (Fig. 5B). NK cell monotherapy exerted some tumor response on the first week of treatment, but nearly half of the animals started to relapse after day seven (Fig. 5C). Addition of virus backbone seemed to add some tumor control benefit to NK cell therapy tumor response, however, therapy relapse was also observed in a few animals (Fig. 5D). Animals receiving vIL-2 virus plus NK cell therapy presented best tumor control with all animals responding to the therapy until the end of the experiment (Fig. 5E). Overall, the combination of vIL-2 virus with NK cell therapy provided statistically significant better tumor control than mock (*p* < 0.001) and NK cell monotherapy (*p* < 0.05) (Fig. 5F).

Regarding lymphocyte biodistribution on day 12, no statistical differences were observed in the levels of NK+ cells present in blood and tumors of any of the experimental groups (Fig. 5G). Curiously, the percentage of CD4+ T cells was statistically significant higher in the blood of mock, NK cell monotherapy, and backbone virus plus NK cells groups than in their respective tumors, while no difference was remarked in the combination with vIL-2 virus (Fig. 5H). In contrast, mock and all treatment groups had statistically higher percentage (*p* < 0.05) of CD8+ T cells detected in blood than in tumors (Fig. 5I).

### Anti-tumor control in NK cell plus vIL-2 virus therapy is associated with increased TIL cytotoxicity

**Fig. 6:**
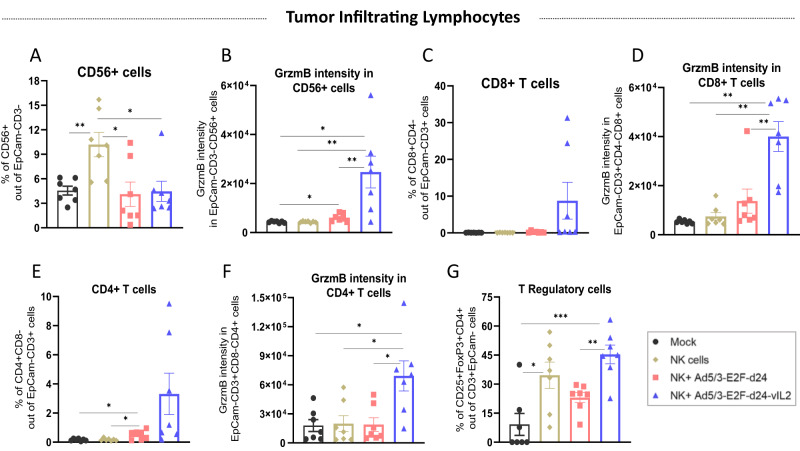
Immune studies of tumor infiltrating lymphocytes in treated ovarian cancer PDX tumors. On day 12, experiment was finished and tumors were processed into single cell suspension and later analysed by flow cytometry. **A** Intratumoral levels of NK+ cells in percentage. **B** Granzyme B MFI in NK+ cells in tumors. **C** Percentage of CD8+ T cells. **D** Granzyme MFI in CD8+ T cells. **E** CD4+ T cells levels in tumors. **F** Granzyme B MFI in CD4+ T cells. **G** Percentage of TReg cells in treated tumors. Data sets were analysed for statistical significance by unpaired *T* test with Welch´s correction and presented as mean ± SEM. *p < 0.05, **p < 0.01, ***p < 0.001.

To better understand how the interplay between combination therapy and immune cells elicited anti-tumor response, we analyzed the lymphocytes present in tumors collected on day 12. Presence of NK+ cells was significantly higher (*p* < 0.05) in the NK cell monotherapy than in the other studied groups (Fig. 6A). However, only in the combination therapy with vIL-2 virus, NK+ cells presented statistically significant higher (*p* < 0.05) GrzmB intensity over the other treatments and mock (Fig. 6B). In terms of PD-1 intensity in NK+ cells, we observed a trend in the upregulation of PD-1 expression in the vIL-2 virus plus NK cell therapy group, although not statistically significant (Supplementary Fig. 5). Curiously, CD8+ T levels in tumors showed no significant difference among groups, although some tumors in the vIL2 group had a high proportion of CD8+ T cells (Fig. 6C). Conversely, GrzmB MFI in CD8+ T cells was more abundant when the NK cells were used in conjunction with vIL-2 virus, with statistical significance (*p* < 0.05) difference found against the other groups (Fig. 6D).

Additionally, we also examined the CD4+ T cell compartment, and only in backbone virus plus NK cells treatment a significant difference was noted compared to mock and NK cell monotherapy groups, although in some tumors of the vIL-2 virus plus NK cells group had high cell counts (Fig. 6E). Comparably to the other effector lymphocytes, cytotoxic CD4+ T cells were statistically significant highest (*p* < 0.05) in tumors treated with vIL-2 virus and NK cell combination therapy (Fig. 6F). Lastly, levels of TReg cells infiltrating tumors were evaluated and shown to be statistically significant higher in the vIL-2 plus NK cell group (Fig. 6G). NK cell monotherapy had statistically significant more elevated levels of TReg cells than the mock group (*p* < 0.05).

## Discussion

Development of large scale clinical-grade NK cell production and the diversity of allogeneic cell sources used for NK cell generation have supported advances in pre-clinical and clinical studies using NK cells for cancer immunotherapy [4, 5, 10, 11]. Nevertheless, enhancement of NK cell anti-tumor activity for adoptive transfer for treatment of solid tumors remains a challenge. Current therapies focus on potentiating NK cell activation or engagement with target cancer cells, like with use of stimulatory cytokines or bispecific and trispecific engager molecules, respectively [28–31].

From the immunotherapeutic perspective, these strategies do not provide alternatives for circumventing tumor microenvironment immunosuppression, which is a common hindrance of NK cell therapy success in solid tumors [32]. We have previously demonstrated that an adenovirus encoding a human variant IL-2 was capable of counteracting tumor immunosuppression in a hamster pancreatic model as a monotherapy [18]. Moreover, the vIL-2 virus has demonstrated to be safe as a monotherapy, and its backbone has been detected in high concentration in tumors upon systemic administration [18, 33]. In the present study, we propose the use of vIL-2 virus, Ad5/3-E2F-d24-vIL2, as a combination strategy to improve therapeutic response of allogeneic NK cells for the treatment of human OvCa tumors.

OvCa is the deadliest gynecological cancer, it presents high rates of tumor recurrence associated with poor 3-year overall survival, and over two thirds of patients already present an advanced stage of the disease at the time of cancer diagnosis [14, 34, 35]. Actually, in our study, most patients presented tumors at stage IVB, when the disease has spread beyond the organs in the abdomen [35]. Likewise, most of specimens collected, 9 out of 12, were derived from metastatic lesions. From the TME point of view, OvCa is a highly immunosuppressive tumor type characterized by the presence of T regulatory cells, MDSCs, and TAM that promote tumor growth and release of anti-inflammatory agents in the TME [13].

Nevertheless, baseline TME or initial histological state were not an impediment for efficient response in OvCa tumor digests. In fact, our results demonstrate that addition of Ad5/3-E2F-d24-vIL2 virus bolstered NK cell therapy killing effects in a set of OvCa human samples in ex vivo co-cultures regardless of tumor diagnosis or stage. Notably, we also observed better cancer control when the adenovirus was loaded with the vIL-2 cytokine transgene compared to its backbone counterpart in those co-cultures. Such results can be attributed to the selective mode of action of vIL-2 cytokine has on IL-2R of NK+, CD4+ T and CD8+ T cells triggering cell stimulation, compared to inactive effect in TReg cells [36]. Importantly, when said variant is expressed by an engineered oncolytic adenovirus, Ad5/3-E2F-d24-vIL2, additional downregulation of genes associated with MDSCs function is also observed [18], which makes this vectored viral approach particularly appealing for treatment of immunosuppressive tumors like OvCa. Of note, the impedance system used here to evaluate cell cytotoxicity has some limitations to differentiate adherence signals derived from cancer cells and immune cells, such as myeloid cells and NK cells. For this reason, the interpretation of some results like the ones obtained in ex vivo co-cultures of HUSOV5 and HUSOV15 can be difficult. On this regard, future studies investigating cancer cell molecular death like TUNEL staining might help to elucidate this matter.

Corroborating this notion, our analysis of immune cells in tumor co-cultures treated with vIL-2 virus and NK cell therapy showed increased levels of cytotoxic NK+ and CD8+ T cells, while no significant changes were observed in TReg cells. Interestingly, these findings differ from the analysis made in tumors from the OvCa PDX in vivo experiment, where significantly higher levels of TReg cells were found in vIL-2 virus plus NK cells treated animals compared to backbone control and mock groups. Perhaps explaining this finding, our vIL-2 virus therapeutic approach, similarly to other modified IL-2 cytokine candidates, does not prevent expression of wt IL-2 cytokine expression by the activated host immune cells, as well as it does not block the usage of wt IL-2 cytokine by immune cells, including TReg cells present in the TME [18, 37, 38]. Instead, our virus vector continuously produces high levels of vIL-2 cytokine in the TME, as previously demonstrated, that will be taken up by effector cells only, diminishing the overall TReg cell-derived immunosuppression [18]. Expression of wt IL-2 in healthy organs would be interesting to be evaluated in future studies.

Of note, our oncolytic adenovirus vector used to encode vIL-2 cytokine represents a therapeutic advantage for TME remodeling. Modifications made on the adenovirus structure have been optimized to promote increased virus infectivity and amplification in OvCa cells as well as to efficiently lyse cancer cells upon virus infection [22, 23]. In fact, adenovirus-mediated immunogenic cell death is an important mechanism for shedding of pro-inflammatory signals in the TME, such as cell danger signaling molecules, and subsequent engagement of the host immune system with anti-tumor response [16, 18, 39]. Overall, this goes in line with our findings in virus backbone treated groups, where partial control of OvCa progression ex vivo and in vivo can be linked to direct cancer cell debulking and immune response onset. Despite described benefits, our results demonstrate that only when loaded with vIL-2 transgene, the virus consistently reshapes the TME towards a pro-inflammatory state. These results corroborate with previous findings with vIL-2 virus treatment as a monotherapy, where high expression of *CCL2, TNF-α,* and *IL-1β* genes were detected in treated tumors [18]. Unfortunately, due to small tumors sizes, especially in vIL-2 virus combination group, the cytokine profiling study could not be performed.

Another interesting aspect noticed in OvCa co-cultures was the high intensity of CD158b in NK+ cells in the combination therapy. CD158b is part of the family of killer cell immunoglobulin-like receptors (KIR), a group of transmembrane proteins that modulate NK cell cytotoxicity particularly through inhibitory signaling interaction with HLA-ABC receptors [1, 40]. In the cancer context, CD158b upregulation has been negatively associated with NK cell activation and production of CD107, IFN-γ, and perforin even when tumors are exposed to exogenous IL-2 and IL-15 cytokines [40, 41]. In our co-cultures, we hypothesize that augmented CD158b intensity in NK+ cells could indicate a progressive transition of NK+ cells from a cytotoxic to a baseline state, in view of the time point selected for immune cells analysis and the efficient cell killing by the combination approach already observed at earlier hours. However, future studies should investigate the potential effect of the virus backbone and vIL-2 virus monotherapies might have on the modulation of CD158 molecule in NK cells present in OvCa tumors.

Enabling the full potential of adoptive NK cell therapy in vivo represents one of the main current challenges for NK cell therapy success. Here, we demonstrated that vIL-2 virus efficiently increased allogeneic NK cell anti-tumor control in an OvCa PDX mouse model. Animals receiving the combination of vIL-2 virus plus NK cells had the best tumor control compared to the other experimental groups. Such improvement was associated with the ability of the vIL-2 transgene to enhance the cytotoxic potential of NK+ cells, CD4+T, and CD8 + T infiltrating the immunosuppressive OvCa TME. Altogether, these results confirm our ex vivo findings and endorse the therapeutic advantage of using our vIL-2 virus candidate to potentiate allogeneic adoptive NK cell therapy for the treatment of OvCa tumors. Of note, differences on proportion of detected CD4 + T and CD8+ T cells in the ex vivo and in vivo studies can be partially explained by the PBMCs expansion protocol utilised in the latter case prior mice injection. Addition of exogenous wt IL-2 cytokine to cell cultures can condition T cells response more promptly to further cytokine exposure [25, 42]. While in the OvCa ex vivo co-cultures, no cytokine stimulation was done prior the treatment with the combination therapy.

From the NK cell immunotherapeutic perspective, continuous production of vIL-2 cytokine by the adenovirus vector is determinant for sustained NK cell anti-tumor response, although no increase on the NK cell proportions was observed at the time point studied. Possibly due to the regular NK cell life-spam programming after response to target cancer cells [43]. In our in vivo animal experiment, a single dose of allogeneic NK cells was sufficient to promote continued tumor response when cell therapy was used in conjunction with vIL-2 virus. In contrast, in an iPSC-derived NK cell therapy, best anti-tumor response was obtained when a total of 3 doses of NK cells were administrated together with 5 doses of IL-2 cytokine injections into pre-irradiated mice bearing OvCa tumors [8].

Similarly, multiple doses of CD34+ hematopoietic progenitor cell (HPC)-derived NK cells and IL-15 were given to animals bearing OvCa tumors treated with gemcitabine to improve tumor response [11]. Taken together, the data presented here highlights the prospective potential of our vIL-2 virus candidate to unleash the therapeutic potential of NK cells for OvCa treatment, by allowing optimized use of NK cells, with reduced rounds of cell transfer and absence of need for exogenous cytokine therapy. In the clinical context, the latter is particularly relevant in view of eventual constraints with NK cell availability for adoptive transfer and frequent toxicity associated with systemic administration of human stimulatory cytokines [5, 19].

Considering the key role NK cells have in the clearance of viral infections, proposing an adenovirus candidate as a combination strategy for NK cell adoptive therapy could seem like a counter-intuitive approach. In our results, however, the administration of said therapies together resulted in improved cancer cell killing and control of treated OvCa tumors. Of note, adenoviruses possess their own mechanisms for immune evasion, in particular, the E3 region hosts genes closely associated with expression of immunoregulatory proteins such as the E3/glycoprotein19K that binds to MHC I in the endoplasmic reticulum preventing the antigen presentation of viral peptides on the cell surface and activation of CD8+ T cells [6, 44].

To escape NK cell response, adenovirus 5 acts by downregulating co-stimulatory proteins MICA/MICB, CD112, and CD155 and upregulates HLA-E: a non-classical HLA with negative effects on NK cell activation [6, 44, 45]. Importantly, the partially deleted E3 region in the vIL-2 virus construct is replaced by the vIL-2 cytokine transgene, which in turn should facilitate the recognition of virus-infected cells by the host´s lymphocytes. Challenging these expectations, the vIL-2 virus downregulated MHC I (HLA-ABC) intensity in most of the samples studied. CD155 and MICA/MICB values oscillated up and down, while HLA-E intensity remained unchanged in infected OvCa tumor digests. This data suggests that absence of immunoregulatory protein E3/glycoprotein19K is not sufficient to evade NK cells recognition and activation upon vIL-2 virus infected OvCa cells.

In conclusion, our results demonstrate that Ad5/3-E2F-d24-vIL2 is a powerful approach for enabling allogeneic NK cell therapy. Ad5/3-E2F-d24-vIL2 efficiently counteracted the immunosuppressive human OvCa TME by enhancing NK cell and T lymphocyte cytotoxicity, while maintaining tumor-infiltrating TReg levels comparable to NK cell monotherapy. Of note, this preclinical study paves the way for clinical trials with Ad5/3-E2F-d24-vIL2 in combination with NK cells, as well as NK cells derived products such as CAR-NKs, iPSC-NKs and other forms of engineered NK cell therapy.

### Supplementary information

Supplemental material

## Data Availability

All data generated or analysed during this study are included in this published article [and its supplementary information files].
