# Supplementary material for: Boosting cytotoxicity of adoptive allogeneic NK cell therapy with an oncolytic adenovirus encoding a human vIL-2 cytokine for the treatment of human ovarian cancer
Source: Cancer Gene Ther. 2023 Nov 10;30(12):1679–90. doi: 10.1038/s41417-023-00674-3 (PMC10721546; doi:10.1038/s41417-023-00674-3)
Supplement: Supplementary file 1 — Supplemental material [file 41417_2023_674_MOESM1_ESM.pdf]

Boosting cytotoxicity of adoptive allogeneic NK cell therapy with an oncolytic adenovirus encoding a human vIL-2 cytokine for the treatment of human ovarian cancer

Quixabeira DCA[1,2], Pakola S[1], Jirovec E[1], Havunen R[1,2], Basnet S[1], Santos JM[1,2], Kudling TV[1], Clubb JHA[1,2], Haybout L[1], Arias V[1], Grönberg-Vähä-Koskela S[1,3], Cervera-Carrascon V[1,2], Kerkelä E[4], Pasanen A[5], Anttila M[6], Tapper J[7], Kanerva A[7], and Hemminki A[1,2,3,*]

Supplements

Supplementary Table 1 - Flow cytometry panel				
Antigen	Flurochrome	Clone	Catalogue Number	Provider
EpCAM	FITC	9C4	324204	Biolegend
MICA/MICB	APC	6D4	320908	Biolegend
HLA-ABC	PE-Cyanine 7	W6/32	25-9983-42	ThermoFisher
HLA-E	PE	3D12HLA-E	12-9953-42	ThermoFisher
CD155 (PVR)	Alexa Fluor®	SKII.4	337630	Biolegend
CD112 (Netin)	PerCP-Cyanine 5.5	TX31	337416	Biolegend
Propidium Iodide Solution	PI	RUO	421301	Biolegend
CD3	AF700	SK7	344822	Biolegend
CD4	BV570	RPA-T4	300534	Biolegend
CD8	BV510	RPA-T8	563256	BD
PD-1	APC-Cy7	EH12.2H7	329922	Biolegend
CD25	BV711	M-A251	356138	Biolegend
FoxP3	PE-Dazzle	206D	320126	Biolegend
GrzmB	PE	GB11	561142	BD
EpCam	PE-Dazzle	9C4	324232	Biolegend
CD56	BV510	HCD56	318340	Biolegend
CD158b	FITC	DX27	312604	Biolegend

Supplementary Table 1. List of Antibodies and fluorochromes used for the cell immune and MHC studies.

Supplementary Table 2 - IHC antibody panel		
Antibody	Dilution used	Provider
CD4 (104R-16)	1:100	Cell Marque
CD8 (NCL-CD8-4B11)	1:50	N-C
CD56 (156R-96)	1:500	Cell Marque
PD-L1 (741-4860)	Ready to use	Roche

Supplementary Table 2. List of antibodies used for the immunohistochemistry staining of OvCa slides.

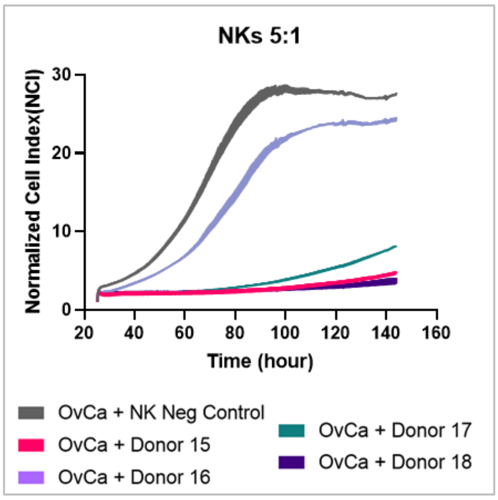

Supplementary Figure 1. NK cytotoxicity screening using healthy blood donors. Ovarian cancer patientderived cell line was seeded and incubated for 24 hours, when freshly isolated and expanded allogeneic NK cells from four different donors were added in 5:1 ratio (E:T). Non-expanded and non-activated NK cells were used as a negative control group.

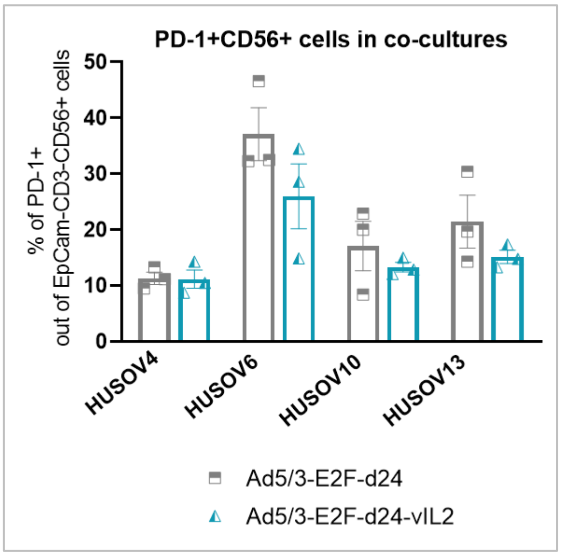

Supplementary Figure 2. Immune status of lymphocytes present in human ovarian cancer co-cultures treated with vIL-2 in combination with adoptive NK cell therapy. HUSOV4, HUSOV6, HUSOV10, and HUSOV13 patient samples were seeded in triplicates at the concentration of $3.5x10^5$ cells per well. After 24 hours incubation period at 37°C, ovarian cancer samples were treated with Ad5/3-E2F-d24-vIL2 virus or Ad5/3-E2F-d24 (100vp/cell). Co-cultures were incubated for 6 more days, then cells were harvested and lymphocytes stained with antibody flurochrome-conjugated for flow cytometry analyses. Percentage of PD-1+CD56+ cells in OvCa co-cultures treated either with Ad5/3-E2F-d24 virus or Ad5/3-E2F-d24-vIL2 virus. Data sets were analysed for statistical significance by unpaired T test with Welch´s correction and presented as mean +- SEM.

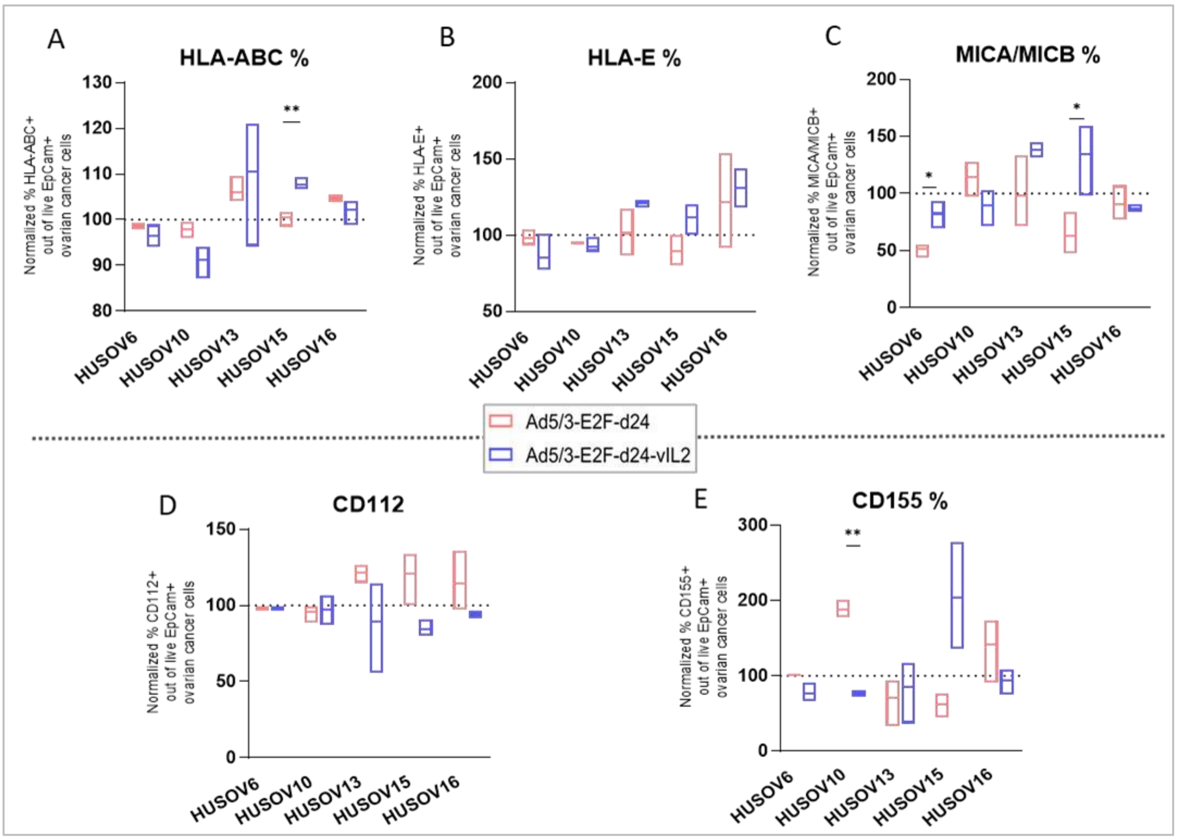

Supplementary Figure 3. Differences on the expression of (A) HLA-ABC, (B) HLA-E, (C) MICA/MICB, (D) CD112, and (E) CD155 percentage in ovarian cancer cells from patient samples (HUSOV6, HUSOV10, HUSOV13, HUSOV15, and HUSOV16) upon Ad5/3-E2F-d24-vIL2 virus infection. Results were normalized to their respective mock (uninfected) group. *p<0.05, **p<0.01.

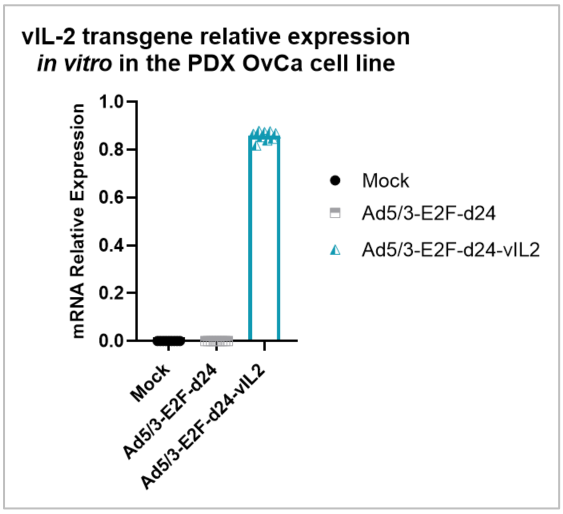

Supplementary Figure 4. *In vitro* relative mRNA expression of vIL-2 cytokine transgene in the OvCa PDX cell line used for the in vivo animal experiment. OvCa PDX cells were plated in triplicates and after 24 hours of incubation were infected either with Ad5/3-E2F-d24 virus or Ad5/3-E2F-d24-vIL2. Mock control group was left uninfected. OvCa PDX cells were harvested after 3 days of virus infection and RT-qPCR performed in triplicates as described before(18). Primers, probes, and qPCR cycling conditions have been described previously(18).

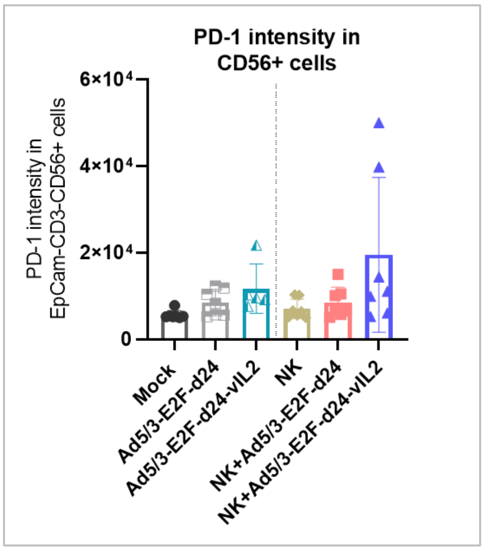

Supplementary Figure 5. Immune studies of tumor-infiltrating lymphocytes in treated ovarian cancer PDX tumors. On day 12, the experiment was finished and tumors were processed into single-cell suspension and later analysed by flow cytometry. Levels of PD-1 MFI in NK+ cells in tumors treated with virus backbone and vIL-2 virus monotherapy treatments, and the respective viruses in combination with NK cell therapy strategy. Data sets were analysed for statistical significance by unpaired T test with Welch´s correction and presented as mean +- SEM.
